# Transportable, portable, wearable and (partially) implantable haemodialysis systems: comparison of technologies and readiness levels

**DOI:** 10.1093/ckj/sfae259

**Published:** 2024-08-24

**Authors:** Fokko P Wieringa, Dian Bolhuis, Henning Søndergaard, Stephen R Ash, Cian Cummins, Karin G F Gerritsen, Jeroen Vollenbroek, Tugrul Irmak

**Affiliations:** IMEC the Netherlands - Health Research, Eindhoven, The Netherlands; UMC Utrecht - Nephrology Dept, Utrecht, The Netherlands; European Kidney Health Alliance - WG3, Brussels, Belgium; IEC - TC62D/MT20, Geneva, Switzerland; UMC Utrecht - Nephrology Dept, Utrecht, The Netherlands; European Kidney Health Alliance - WG3, Brussels, Belgium; Danish Kidney Association, Copenhagen, Denmark; HemoCleanse, Inc. and Ash Access Technologies - R&D, 3601 Sagamore Parkway North, Lafayette, IN, USA; IMEC, Leuven, Belgium; UMC Utrecht - Nephrology Dept, Utrecht, The Netherlands; University Medical Center Utrecht - Pathology, Utrecht, The Netherlands; IMEC the Netherlands - Health Research, Eindhoven, The Netherlands; UMC Utrecht - Nephrology Dept, Utrecht, The Netherlands; UTwente, Enschede, The Netherlands; UMC Utrecht - Nephrology Dept, Utrecht, The Netherlands; European Kidney Health Alliance - WG3, Brussels, Belgium

**Keywords:** kidney replacement therapy, miniaturization, mobility, technology readiness level, trade-offs

## Abstract

**Background:**

Dialysis modalities and their various treatment schedules result from complex compromises (‘trade-offs’) between medical, financial, technological, ergonomic, and ecological factors. This study targets summarizing the mutual influence of these trade-offs on (trans)portable, wearable, or even (partially) implantable haemodialysis (HD) systems, identify what systems are in development, and how they might improve quality of life (QoL) for patients with kidney failure.

**Methods:**

HD as defined by international standard IEC 60601–2-16 was applied on a PUBMED database query regarding (trans)portable, wearable, and (partly) implantable HD systems. Out of 159 search results, 24 were included and scanned for specific HD devices and/or HD systems in development. Additional information about weight, size, and development status was collected by the internet and/or contacting manufacturers. International airplane hand baggage criteria formed the boundary between transportable and portable. Technology readiness levels (TRLs) were assigned by combining TRL scales from the European Union and NATO medical staff.

**Results:**

The query revealed 13 devices/projects: seven transportable (six TRL9, one TRL5); two portable (one TRL6–7, one TRL4); two wearable (one TRL6, one frozen); and two partly implantable (one TRL4–5, one TRL2–3).

**Discussion:**

Three main categories of technical approaches were distinguished: single-pass, dialysate regenerating, and implantable HD filter with extracorporeal dialysate regeneration (in climbing order of mobility).

**Conclusions:**

Kidneys facilitate mobility by excreting strongly concentrated waste solutes with minimal water loss. Mimicking this kidney function can increase HD system mobility. Dialysate-regenerating HD systems are enablers for portability/wearability and, combined with durable implantable HD filters (once available), they may enable HD without needles or intravascular catheters. However, lack of funding severely hampers progress.

## INTRODUCTION

Chronic kidney disease (CKD) and its end stage—kidney failure—are a steadily growing worldwide problem [1, 2]. The field of kidney replacement therapy (KRT) is facing an innovation paradox [3], of which most people are unaware: a widely spread—but unfortunately wrong—public perception is that health problems for patients with chronic kidney failure are ‘solved’ by dialysis or kidney transplantation. This misperception has caused severe ‘underfunding’ of appropriate research for over 50 years [4]. The grim present reality is that 5-year survival on dialysis is worse than for most cancer types, even though the general public and most policy makers typically perceive cancer as far more lethal/impactful [5, 6].

**Figure 1:**
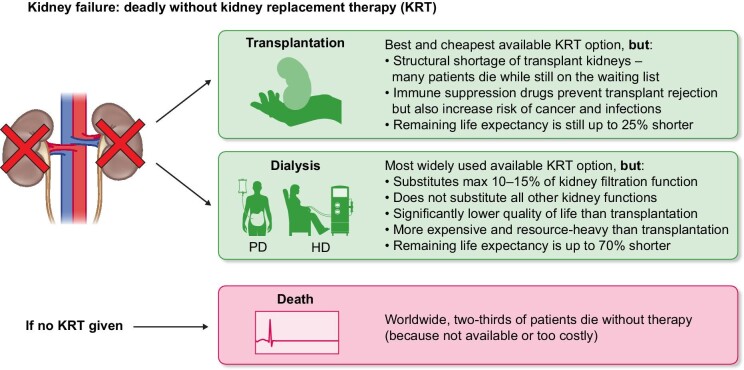
Main categories of currently available KRT: transplantation, PD, and HD. From these three forms of KRT, HD is the most widely applied (and the most expensive). Without some form of KRT, kidney failure is deadly. Globally, an alarming amount of people with kidney failure have no access to KRT because it is either not available in their region, or they cannot bear the costs of treatment.

Figure 1 summarizes the main categories of currently available KRTs to keep patients with kidney failure alive. Although transplantation is the best (and least expensive) option, there is a persistent lack of suitable donor kidneys [7]. Also, not all patients are suitable for transplantation [8, 9]. Those who are lucky enough to receive a donor kidney must take immunosuppressive drugs to prevent rejection. This increases the risk of malignancy and infection, and decreases (or sometimes even removes) the effectiveness of vaccines [10].

Most patients who receive KRT are on a form of dialysis, which is subdivided into two main categories: peritoneal dialysis (PD) and haemodialysis (HD). HD is mostly performed in-centre, although stimulating home HD (HHD) looks favourable for increasing QoL, while decreasing costs for society as a whole. But the adaptation of HHD (and type of HHD treatment schedule) strongly depends on national reimbursement structures [11]. If patients with kidney failure live in an area where KRT is either not available at all or too expensive for them to afford, they die [12]. Note that this grim reality is faced by a shocking two-thirds of all kidney failure patients worldwide [1, 2].

Yet, highly developed countries also feel a heavy economic burden from the cost of dialysis (especially HD, which is more costly than PD), which is steadily growing towards becoming unsustainable [2].

The *Clinical Kidney Journal* editorial team invited us to contribute a narrative review regarding transportable, portable, wearable, and (partly) implantable HD systems intended for use at home or during daily life. Hence, we will furthermore focus on HD.

An important motivation to compose this article is that quality of life (QoL) is low in dialysis patients. Using the WHOQOL-BREF questionnaire, patients treated with HD were found to score lower in QoL compared to a gender- and age-matched control group [13]. In fact, QoL was found to be low in all patients with CKD using the KDQOL questionnaire, but especially in patients treated with dialysis [14, 15]. Therefore, improvement of QoL should be strongly considered when developing new HD modalities.

Portable and wearable devices could lead to this improvement, in several ways; first of all, by an increase in mobility. Recently, Wilson *et al*. created a survey to examine patient preferences in kidney replacement therapies, in which patients were asked to choose one out of two treatment options with different risk and benefit profiles [16]. They found that total mobility weighed heavily as a benefit in decision-making. However, patients would be willing to only increase the risk of infection and death by 1% and 0.5% maximum, respectively, if that would mean that they would be given total mobility during treatment without being attached to a machine. This illustrates the importance of improving mobility from a dialysis patient's perspective, but not when the risks involved are considerably higher compared to conventional treatments.

Note that QoL comprises multiple strongly intertwined domains: somatic, psychological, social, and environmental. In a qualitative study in which caregivers from patients with end-stage kidney disease were asked about important components for the QoL of these patients, three main categories were found, one of which was social disruption [17]. The social domain of HD patients is affected by lack of autonomy and/or mobility and one might thus assume that a portable or wearable device can positively affect QoL.

It is crucial to include patients in design teams and help to unite their voice worldwide towards policy makers and innovators. To this intent, the American Association of Kidney Patients started the ‘Decade of the Kidney^TM^’ initiative, which was joined by the European Kidney Patients Federation (EKPF) and European Kidney Health Alliance (EKHA) to jointly stimulate the realization of innovative kidney replacement therapies [18].

This article considers:

• Several fundamental trade-offs and limitations that influence how mankind can construct (trans)portable, wearable, or even (partly) implantable HD systems.
• Various embodiments of such HD systems, either already on the market, or initiatives thereto that are reported on within scientific literature.
• The technology readiness level (TRL) of these systems (and the TRL concept itself).
• How increasingly mobile HD systems might improve the QoL for patients globally.

## METHODS

We applied the definition of HD as given by the international standard IEC 60601–2-16, describing requirements for the safety and essential performance of HD devices, which is as follows: ‘Process whereby concentrations of water-soluble substances in a patient's blood and an excess of fluid of a patient are corrected by bidirectional diffusive transport and ultrafiltration across a semi-permeable membrane separating the blood from the dialysis fluid’ [19].

To look for HD systems matching the thus defined scope of our narrative review, we searched the PUBMED database using the following query: {[portable(Title/Abstract)] OR [wearable(Title/Abstract)] OR [implantable(Title/Abstract)] AND [haemodialysis(Title/Abstract)] OR [haemodialysis(Title/Abstract)] OR artificial kidney} with a search time window from 1 January 2021 to 19 December 2023 (date of search). This query revealed 159 initial hits of which 24 articles focused on transportable, portable, wearable, or (partly) implantable HD devices or research projects developing such devices. For details regarding the inclusion/exclusion criteria and query evaluation, see the Supplementary Data.

Among the specific HD systems mentioned in the included articles, distinction between ‘transportable’ and ‘portable’ was made using the following rationale: Regarding travelling, the most restrictive rules for size and weight of baggage apply to air transport. For passengers, the most relevant aspect is whether a baggage item falls into the category ‘odd-size check-in’ (more costly and time consuming), ‘regular size check-in’, or ‘carry-on’. The Danish Kidney Association contributed preferences of home HD patients with a lot of experience in air travel. They considered baggage that needs to be checked-in and stowed in the cargo area as only ‘transportable’, whereas to them only carry-on luggage is truly ‘portable’. The International Air Transport Association (IATA) states that

‘carry-on baggage allowance can vary according to the airline, the cabin class you are travelling in and even the size of the aircraft. As a general guide, carry-on baggage should have maximum length of 22 in (56 cm), width of 18 in (45 cm) and depth of 10 in (25 cm). These dimensions include wheels, handles, side pockets, etc.’ [20].

For this study, we thus reasoned that, from the patient perspective, portable should imply that a packed HD device should meet this IATA recommendation, because carry-on baggage is much less likely to get damaged or lost during handling, which is paramount for patients. The trip duration will of course influence how many sets of consumables should be taken along. We reasoned that the HD device itself might be packed into one carry-on case, and a reasonable number of consumable sets into another carry-on case. It is not unusual to arrange for on-destination delivery of additional sets, but one wants to make sure to at least be able to treat on arrival and bridge some time in case delivery of extra sets is delayed.

As an indication of the development status for the various identified devices, we assigned a TRL to the listed HD systems, using the combined criteria of the two TRL scales listed in Table 1. In addition to the TRL scale applied by the European Union within their calls for research proposals, we also added the TRL scale applied by the medical staff of NATO (because portability, ruggedness, and ease of use are strongly pronounced in that TRL scale) [21, 22].

**Table 1:** Applied TRL scales, used by the EU and NATO medical staff.

	Horizon Europe NCP Portal	
TRL	TRL Self-Assessment Tool [22]	NATO TRL Scale for Medical Devices [21]
1	Basic principles observed	Basic Principles Observed and Reported in Context of a Military Capability Shortfall Lowest level of technology readiness. TRL 1 Decision Criterion: Scientific literature reviews and initial Market Surveys are initiated and assessed. Potential scientific application to defined problems is articulated.
2	Technology concept formulated	Technology Concept and/or Application Formulated Invention begins. Intense intellectual focus on the problem with generation of scientific ‘Paper Studies’ that review and generate research ideas, hypothesis, and experimental designs for addressing the related scientific issues. TRL 2 Decision Criterion: Hypothesis(es) generated. Research plans and/or protocols are developed, peer reviewed, and approved.
3	Experimental proof of concept Initial proof of concept demonstrated with a limited number of *in vitro* and *in vivo* trials including the expected device characteristics	Analytical and Experimental Critical Function and/or Characteristic Proof of Concept Active research and development are initiated. Basic research, data collection, and analysis begin to test hypothesis, explore alternative concepts, and identify and evaluate component technologies. Initial tests of design concept, and evaluation of candidate(s). TRL 3 Decision Criterion: Study endpoints defined. Animal models (if any) are proposed. Design verification, critical component specifications and tests (if a system component, or necessary for device Test and Evaluation) developed.
4	Technology validated in a laboratory Proof of concept and safety of the device is demonstrated *in vitro*, *ex vivo*, or *in vivo* conditions (non-GMP, Good Manufacturing Practice) System components integrated and tested regarding preliminary efficiency and reliability	Component and/or Breadboard Validation in Laboratory/Field Environment Basic technology components are integrated to establish that they will work together. Non-GLP laboratory research to refine hypothesis and identify relevant parametric data required for technological assessment in a rigorous (worst case) experimental design. Exploratory study of candidate device(s)/systems (e.g. initial specification of device, system, and sub-systems). Candidate devices/systems are evaluated in laboratory and/or animal models to identify and assess potential safety problems, adverse events, and side effects. Procedures and methods to be used during nonclinical and clinical studies in evaluating candidate devices/systems are identified. TRL 4 Decision Criterion: Proof of concept demonstrated for candidate devices/systems and laboratory/animal models defined. Initial device master record completed.
5	Technology validated in relevant environment (industrially relevant environment in the case of key enabling technologies) Pre-clinical studies including GLP animal safety and toxicity. GMP manufacturing process and quality controls identified. Classification of the device by appropriated regulatory body established. Accreditation when appropriate initiated	Component and/or Breadboard Validation in a Relevant (Operating) Environment Fidelity of sub-system (breadboard) representation increases significantly. Further development of selected candidate technologies. Devices compared to existing modalities and indication for use and equivalency demonstrated in model systems. Examples include devices tested through simulation in tissue or organ models, or animal models if required. All component suppliers/vendors are identified and qualified. Vendors for critical components audited for GMP compliance. Component tests, component drawings, and device master record verified. Product development plan drafted. TRL 5 Decision Criterion: Regulatory authorities have reviewed submissions of required data to determine if clinical trials may proceed.
6	Technology demonstrated in relevant environment (industrially relevant environment in the case of key enabling technologies) Medical device prototype demonstrated in operational environment. Clinical testing and safety demonstrated Required accreditation in progress	System/Sub-System Model or Prototype Demonstration in a Realistic (Operating) Environment or Context Representative model or prototype system, which is well beyond the representation tested for TRL 5, is tested in a more realistic laboratory or simulated operational environment. Represents a major step up in a technology's demonstrated readiness. First Phase clinical trials conducted to demonstrate the safety of the candidate device in a small number of humans under carefully controlled and intensely monitored clinical conditions. Validation of the master plan for critical components and final device assembly. Production technology demonstrated through production-scale GMP plant qualifications. TRL 6 Decision Criterion: Data from First Phase trials meet national clinical safety requirements and support proceeding to next phase of clinical studies.
7	System prototype demonstration in operational environment Medical device final product design is validated Final prototypes intended for commercialization use produced and tested When applicable, accreditation completed	System Prototype Demonstration in an Operational Environment or Context (e.g. Exercise) Prototype near or at planned operational system level. Represents a major step up from TRL 6, requiring the demonstration of an actual system prototype in an operational environment, such as in a relevant platform or in a system-of-systems. Second Phase clinical effectiveness and safety trials are conducted with a fully integrated device prototype in an operational environment. Continuation of closely controlled studies of effectiveness and determination of short-term adverse events and risks associated with the candidate product. Functional testing of candidate devices is completed and confirmed, resulting in final selection of prototype device. Regulatory agencies have approved continued development and testing. TRL 7 Decision Criterion: Second Phase clinical effectiveness and safety trials are completed. Final product design is validated, and final prototypes and/or initial commercial scale devices are produced. Data is collected, presented, and discussed with regulatory agencies. Agencies support continued development. Clinical endpoints and test plans agreed to by regulatory agencies. Next Phase clinical study plan is approved.
8	System complete and qualified Manufacturing process validated Pre-market application submitted and approved for medical device Device demonstrated in real life conditions, support structure in place for technical problems	Actual System Completed and Qualified through Test and Demonstration Technology has been proven to work in its final form and under expected conditions. Implementation of expanded controlled and uncontrolled Phase 3 trials to gather information relative to the safety and effectiveness of the device. Trials are conducted to evaluate the overall risk-benefit of using the device, and to provide an adequate basis for product labelling. Process validation completed and followed by lot consistency/reproducibility studies. TRL 8 Decision Criterion: Approval of the device by national or international regulatory authorities.
9	Actual system proven in operational environment (competitive manufacturing in the case of key enabling technologies; or in space) Medical device ready to be acquired by clients and end users	Actual System Operationally Proven through Successful Mission Operations Application of the technology in its final form and under mission conditions. Post-marketing studies (clinical or nonclinical) may be required by national or international regulatory authorities. TRL 9 Decision Criterion: None—continue (post-marketing) surveillance.

These two TRL scales do not conflict, but the European Union (EU) scale provides relatively short telegram-style descriptions, while the NATO scale provides some more useful details that can help to decide between levels when in doubt.

Within the fields of military, aerospace, and the silicon chip industry, the TRL concept is well-known, but far less so in medical technology. It is a useful tool to communicate the progression of new technologies into daily life. Fundamental breakthroughs in medical technologies undoubtedly deserve exciting media coverage, but it would be good if science journalists would consequently add a TRL indicator and explain what it means.

## RESULTS

Table 2 lists the various HD systems (and research projects towards realizing them) that were mentioned in the articles resulting from the literature search.

**Table 2:** Transportable, portable, wearable, and (partly) implantable HD devices, mentioned within the included articles. A TRL value was assigned, using the (per-row combined) criteria listed in Table 1. Note that this list is not exhaustive. The not-for-profit organization Home Dialysis Central also maintains a website with home dialysis devices, which is periodically updated: https://homedialysis.org/home-dialysis-basics/machines-and-supplies/haemodialysis-machines.

Device		Features	Development Stage	Ref(s)
Transportable (odd-size check-in baggage)	VersiHD from NxStage https://www.nxstage.com/hcp/products/nxstage-versihd	36 kg (excl. dialysate bags or water purification unit). 46×38×38 cm (HxWxD) An updated version exists that has an interactive screen offering user guidance (GuideMe software). Max dialysate flow rate 300 ml/min, max ultrafiltration rate 2.4 l/h. Max blood flow rate 600 ml/min. Single-pass system (block diagram Fig. 2).	TRL 9, since 2017 on the market	[23]
	System One (S) from NxStage https://www.nxstage.com/hcp/products/nxstage-system-one-s/	34 kg (S) or 32 kg (One) (for both types: excl. dialysate bags in steps of 5 l, or PureFlow SL water purification unit) 46×38×38 cm (S) or 38×38×38 cm (One) (H×W×D). Single-pass system (block diagram Fig. 2). Designed for short daily low-dialysate-flow home HD. Max dialysate flow rate 200 ml/min (System One, bags) or 300 ml/min (S), max ultrafiltration rate 2.4 l/h. Max blood flow rate 600 ml/min.	TRL 9, on the market for over 15 years. Recently, pulled off German market, due to tight reimbursement.	[23, 24]
	SelfCare + from Quanta https://quantadt.com/uk/	35 kg (excl. dialysate and QDS™ water purification cart). 48×37×45 cm (H×W×D). Max dialysate flow rate 500 ml/min. Single-pass system (block diagram Fig. 2).	TRL 9, CE-mark since 2015, FDA approval since 2021. Recently pulled off EU market.	[24]
	S3 from Physidia https://www.physidia.com/en/your-home-daily-haemodialysis-solution/	24 kg (excluding dialysate bags in steps of 5 L). 40×40×40 cm (H×W×D). Designed for short daily low-dialysate-flow home HD. Max dialysate flow rate 200 ml/min. Max blood flow rate 350 ml/min. Max ultrafiltration rate 1620 ml/h. Single-pass system (block diagram Fig. 2). Can only be used for treatments up to 180 min.	TRL 9, on the market for 10 years.	[23–25]
	DIMI from Infomed. https://www.idialco-infomed.com/en/products/dimi.php	19 kg (excl. 30 kg trolley, excl. max 40 l dialysate) 58×60×63 cm (height x width x depth, without trolley). Dialysate bags (in steps of 20 l). Max dialysate flow rate, substitution flow rate and ultrafiltration rate 200 ml/min. Max blood flow rate 400 ml/min. Applicable for HD, haemodiafiltration, and PD. Single-pass system (block diagram Fig. 2).	TRL 9, has CE-mark and Health Canada approval. FDA approval for home HD pending.	[24]
	PAK from Medtronic (transferred to Mozarc). https://mozarcmedical.com/	Weight and size unknown, but seems larger than IATA guidelines for carry-on hand baggage. Intended for home use. Sorbent (urease-based) dialysate regeneration (block diagram Fig. 2).	TRL 5 (estimated). Medtronic transferred dialysis to the new firm Mozarc in 2023. Device is still in development.	[26, 27]
	Tablo by Outset Medical https://www.outsetmedical.com/tablo/	88 kg, built into a trolley which is also a transport container. 89×45×68 cm (HxWxD). Designed for HD treatments from the ICU to at home. Max dialysate flow rate 300 ml/min. Max ultrafiltration rate 2 l/h. Max blood flow rate 400 ml/min. Single-pass system (block diagram Fig 2).	TRL 9, FDA approval for home HD since 2020.	[23]
Portable	Neokidney PAK from Nextkidney. https://nextkidney.com/	12 kg (excl. 5 l dialysate bag and sorbent cartridge). Meets IATA ‘carry-on’ baggage size limits. Via fistula/shunt or central venous catheter (dual lumen). Designed for low-dialysate-flow home HD. Max dialysate flow rate 300 ml/min, ultrafiltration volume max 2 l/session (bag size limited, currently working on a bag version >2.5 l/session). Max blood flow rate 300 ml/min. Meets IEC Cardiac Floating criteria. Double isolated, thus can be plugged in anywhere (incl. non-grounded outlets) without prior electrical verification (110–240 V, 50–60 Hz). Sorbent (urease-based) dialysate regeneration (block diagram of Fig. 3).	TRL 6–7. Production and human clinical trials ongoing. Presented at ASN 2023 and ERA 2023. CE-mark expected 2025.	[27, 28]
	Dharma™ PAK from EasyDial. Firm Easydial transformed to firm Diality, and product seems renamed to Moda-flx. https://www.diality.com/	6.2 kg (excl. 3.7 l dialysate; from Dharma description). 52×29×18 cm (H×W×D). Sorbent (urease-based) dialysate regeneration (block diagram of Fig. 3).	TRL 4 (*estimated*). In vitro tests Dharma described in 2017 and 2021. Diality presented during ASN 2023.	[27, 29]
Wearable	WAK from Dr Gura. https://drgura.com/contents/wearable-artificial-kidney.php	5 kg weight (incl. dialysate) Wearable on belt, for HD on-the-go. Battery powered. Double-lumen tunnelled central venous catheter. Gambro Polyflux 6H, Baxter dialyser (0.6 m^2^). Sorbent (urease-based) dialysate regeneration (block diagram of Fig. 3).	TRL 6–7. 3 prototype generations, all demonstrated in human clinical trials. Further development in progress. No series production yet.	[26, 27, 30–33]
	MiniKid from Nanodialysis. https://www.nanodialysis.nl/hemodialysis/	2 kg weight (excl. 300 ml dialysate). 25×15×6 cm/31×18×6 cm in shoulderbag (H×W×D). Via fistula/shunt or central venous catheter. Sorbent (nanostructured, nanoporous polymers) and electro-oxidation (block diagram of Fig. 3).	TRL 5. Tested in goats. Project put on hold, to prioritize development of a sorbent-based PD-device.	[33]
Implantable (partly)	Implantable silicon nanopore membrane haemodialyser (SNMHD) from ‘the Kidney Project’ (a cooperation between UCSF $\& $ Vanderbilt Medical School). See note a) https://www.youtube.com/watch?v=CFRtc5V1H-c	Probably <0.5 kg (no exact weight listed). 1.2×10.6×5.7 cm (H×W×D). Implantable HD filter. No blood pump needed (heart provides driving blood pressure) thus no extracorporeal blood circuit needed (block diagram of Fig. 4). Project won several KidneyX prizes.	TRL 4–5, *in* *vivo* established in a well-defined stable uremic animal model (female Yucatan minipig). Iterative improvement cycles on prototypes are ongoing.	[30, 34, 35]
	Implantable SNMHD from the KIDNEW EU-project. See note a) https://www.kidnew.eu/	Probably <0.5 kg (no weight and size listed). Targeting an implantable HD filter with similar modular approach as ‘the Kidney Project’ (no external blood circuit, block diagram of Fig. 4). Upscaling of production methods separately funded by the NXTGEN HighTech Biomed04 project of the Dutch national growth fund. https://www.myscience.org/news/wire/nxtgen_hightech_invests_38_million_in_new_technology_solutions_for_future_kidney_patients-2023-utwente.	TRL 2–3, first SNMHDs *in* *vitro* tested. Module for on-chip wireless power transfer and data link, sensor interfacing, memory, and microprocessor at TRL4.	[34]

a Both partly implantable solutions need an external device to supply the dialysate circuit. A dialysate-regenerating device offers best mobility, although a single-pass HD system could also be applied.

## DISCUSSION OF SOME TECHNICAL TRADE-OFFS THAT INFLUENCE MOBILITY ASPECTS

**Figure 2:**
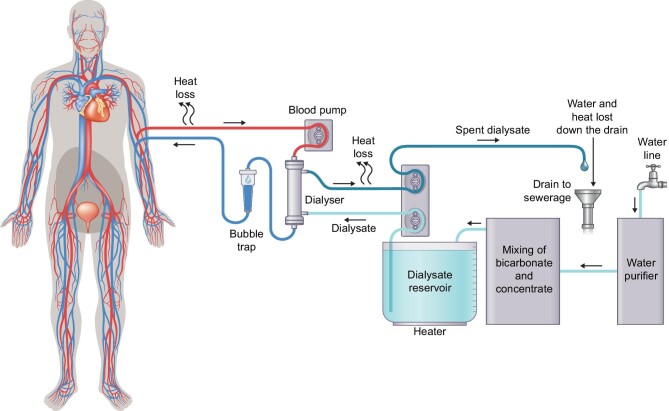
Strongly simplified diagram of a single-pass HD system. After passing the dialyser, the dialysate is discarded into a drain system. This consumes a high volume of dialysate (and energy to warm it up). A drain is needed to dump the spent dialysate (as patients will not be able to manually empty a large volume reservoir). Heat loss occurs in the blood circuit, dialysate circuit and through the drain. Water consumption and energy consumption are thus considerable. Note that water losses via, for example, reverse osmosis water treatment are not even depicted here. When travelling, the water treatment parts usually are left at home. Instead of the dialysate reservoir, dialysate fluid bags (providing the same volume) might be applied. Single-pass HD machines down to a weight of 19 kg are on the market (see Table 2, section ‘transportable’).

**Figure 3:**
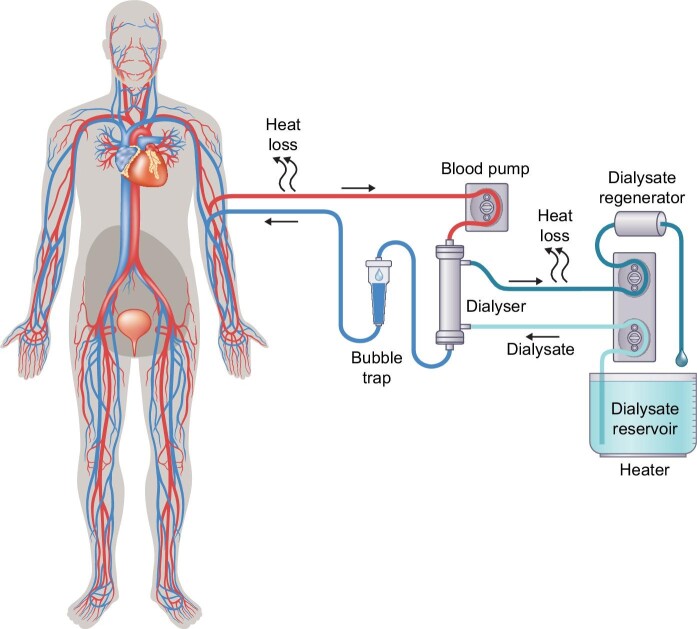
Strongly simplified diagram of a dialysate-regenerating HD system. After passing the dialyser, the dialysate is stripped of toxins and regenerated to the conditions of fresh dialysate in a closed circuit. This drastically reduces the required dialysate volume. The heat loss in the blood circuit remains the same, but the heat loss in the dialysate circuit is far less (once warmed up, only a little energy is needed to maintain temperature) and there is no heat loss into a drain system. This significantly reduces water and energy consumption. A small liquid reservoir can be manually emptied into any existing sink or toilet, thus no connection to a dedicated drain is needed. Weight and size are thus reduced in comparison to Fig. 2. Note that also here the depicted dialysate reservoir might be embodied as a flexible bag. Both portable and wearable embodiments can be found in Table 2.

**Figure 4:**
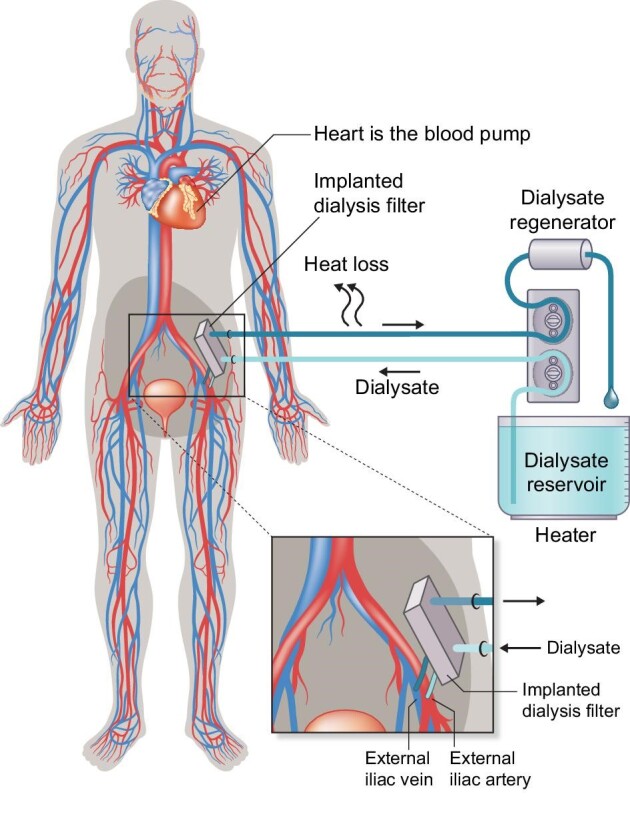
Strongly simplified diagram of a partially implantable HD system without extracorporeal blood circuit. The dialyser is implanted inside the body and perfused continuously, driven by the blood pressure of the heart. Only an extracorporeal dialysate circuit is needed. The same dialysate regeneration method(s) as depicted in Fig. 3 can be applied. Dialysate volume is just as low as described in Fig. 3, but thanks to the implanted dialyser there is no heat loss in the blood circuit, which means that energy consumption is lower than in Fig. 3, which further increases mobility. When not dialysing, the incoming and outgoing dialysate connections are simply short-circuited.

Progression of mobility, from transportable (can be moved, but with some effort), to portable (easy to take along, but stationary during treatment), via wearable (treatment on-the-go possible) to (partly) implantable HD requires significant steps in miniaturization of the HD device itself, as well as its consumables. Mobility also is served by decreased dependence on connections to infrastructure (such as medical grade wall socket outlets, water lines, and drainage plumbing). Next, we discuss the most dominant basic technological trade-offs, supported by Figs 2–4 that show strongly simplified and generalized technical principles to graphically summarize the major steps in mobility.

### The dialysis filter

The functional core of each HD system is the dialysis filter. Portable or wearable HD systems may work well with existing polymer fibre-based HD filters (typically consuming one disposable filter per treatment session) as they have an extracorporeal blood circuit. But for implantation, the present polymer fibre-based filters are unsuitable, as they are prone to clotting within a period of several hours to days (hence, a new filter is usually applied for each session). They also need a blood pump to deliver the high driving pressure to perfuse the thin long fibres (up to 600 mmHg). The technology of the HD filter largely determines the engineering trade-offs for the rest of the HD system. An HD filter suitable for implantation should be very compact, providing at least 15 ml/min filtrate with 400 cm^2^ total filtration area, packed into a ‘fist-size’ device (implying 25–50× reduced area compared to polymeric filters). Ideally, it should be highly permeable for molecules up to nearly the size of albumin (65–66 kD) and then abruptly drop off to zero. It should be extremely resistant to fouling and clotting, and should possess such high porosity, hydraulic permeance, and diffusivity that it can be driven by the natural blood pressure from the heart (see also the section ‘Haemodialysis without an extracorporeal blood circuit’). Compared to the broad Gaussian-like effective pore diameter distribution of present polymer fibre-based HD filters, silicon nanopore membrane (SNM) filters have an inherently sharper defined nearly monodisperse pore diameter, and straight pathways through the pores, instead of the tortuous pathways inherent to polymer fibre-based filters. SNMs thus offer a better trade-off regarding increasing the clearance of middle molecules versus decreasing albumin loss. Implanted membranes also should survive shockwaves from a typical fall or a moderate speed car accident.

### Water and electricity

Water and electricity are important consumables (and strongly interconnected). Let us first look at the water.

Our kidneys enable us to live on the land without permanent access to vast amounts of drinkable water. An adult human can thrive on a daily water intake of ∼1.5–2.5 l in a moderate climate, because the tubules in our kidney nephrons reclaim 98%–99% of the water from our daily glomerular filtrate. Without tubules, we would have to drink that volume to survive. Additionally, that volume should then contain narrow-balanced amounts of nutrients and electrolytes. It is evident that this would dramatically limit our mobility and the areas where we could live.

Yet, HD patients find themselves in an analogue sort of situation: All currently commercially available HD machines do not mimic tubular functions at all and consume vast amounts of water per treatment, which must be precisely mixed with salts to create dialysate. As the Association for the Advancement of Medical Instrumentation (AAMI) published:

‘Haemodialysis and hemodiafiltration can expose the patient to more than 500 l of water per week across the semi-permeable membrane of the hemodialyser or hemodiafilter. Healthy individuals seldom have a weekly oral intake above 12 l. This over 40-fold increase in exposure requires control and monitoring of water quality to avoid excesses of known or suspected harmful substances.’ [36].

Note thus that the large volumes of water, consumed by HD machines, also need to be much better purified than the potable water quality that we normally drink. A typical reverse osmosis water purification system requires ∼3 l of good drinking water to make 1 litre of dialysis water, meaning two-thirds of the consumed drinking water goes down the drain (not depicted in Fig. 2, as also other water purification technologies exist).

But that is not all. A huge amount of electric energy is needed to drive the water purification process and to warm-up all the dialysis water to body temperature. And doing this fast enough to serve the required dialysate flow, requires relatively large heater contact surfaces (which increases capacitive electrical leakage current, typically causing a need for heavily grounded wall socket outlets) [19].

All this is caused by the fact that after having passed through the dialysis filter, the ‘spent dialysate’ is discarded in a drain towards the sewerage system. This so-called ‘single-pass’ principle (see Fig. 2) is applied in all currently marketed HD machines. Some machines reclaim part of the injected energy by a heat-exchanger that pre-warms the incoming fresh dialysate with heat from the spent dialysate before discarding it, but that is all (and a heat-exchanger in turn adds size and weight). Although several commercial HD machines—designed for use at home and transportability for travelling—are on the market, they all are single-pass machines that require a reliable infrastructure for clean water and electric energy (with certified electric grounding for safety) and hooking up to a drain. This hampers mobility and affordability (especially in developing countries).

If, however, one could somehow regenerate the ‘spent dialysate’ so that it becomes as good as new again, this would drastically reduce the consumption of both water and energy (see Fig. 3). Sorbent technology is the oldest—and, so far, only clinically applied—approach to regenerate dialysate in a closed loop, which is currently seeing a revival [36–38]. Also, other promising dialysate regeneration technologies such as electro-oxidation and photocatalytic oxidation are in development (TRL 4–5) [39, 40]. Both these technologies look highly promising (as they might be applied in a reusable embodiment) but need improvements regarding the avoidance of unwanted toxic byproducts of the oxidation process.

### The desire to revive dialysis at home (or even anywhere)

It is noteworthy that in 1972 in the USA 90% of all chronic dialysis patients (counting HD and PD together) did their treatments at home. But after 1972, the reimbursement system in the USA changed, making in-centre dialysis much more lucrative than home dialysis (especially regarding HD) [41–43]. At present, however, several factors are reviving the interest in home dialysis (or even dialysis anywhere):

• An internationally observed increasing shortage of trained dialysis nurses and technicians versus an increasing number of patients.
• The COVID-19 pandemic reminded the world of the almost forgotten infection risk involved in three-times-weekly transporting groups of vulnerable patients into a centre, whereas home-therapy offers far better options to isolate against infection [44–46].
• Kidney patient associations are increasingly raising their voices while internationally joining forces, demanding therapies that can be scheduled around their life, instead of scheduling their life around in-centre treatments [47].
• In at least one case (*Nextkidney*), press releases learned that multiple large health care insurance companies from the Netherlands and France jointly invested in the development of a portable home HD machine, because this would lower costs, while improving patient QoL.

Several countries have published plans to raise the percentage of home dialysis [48–51], but how do we realize such plans? Let us look at one of the biggest hurdles.

### Vascular access for the extracorporeal blood circuit

The Achilles heel of HD—in particular at home—is the need for vascular access to enable the extracorporeal blood circuit needed by all present commercial HD machines. However, extracorporeal blood circuits are only needed because of:

• The sheer size and weight of the HD machine and the HD filter.
• The need for a powerful blood pump to provide considerable pressure needed to perfuse the currently marketed polymer fibre bundle dialysis filters (requiring a matching energy supply towards that blood pump).
• The limited time that currently marketed polymer fibre bundle dialysis filters can be used before they start clotting and must be replaced (or disconnected and cleaned off-line).
• Technical maintenance aspects regarding the machine.
• Microbiological aspects, which drive the use of disposables.

These aspects are graphically summarized in Fig. 2.

What if these (mutually intertwined) problems could be tackled? Figures 3 and 4 illustrate how this might be achieved in a stepwise manner, following the KHI innovation roadmap [48, 52].

The section ‘Water and electricity’ already highlighted how regeneration of dialysate could support mobility. Figure 3 depicts this in a generalized block diagram (note the size reduction compared to Fig. 2).

### Haemodialysis without an extracorporeal blood circuit?

A paradigm shift towards implantable HD filters—as depicted in Fig. 4—would be a true game-changer, but it would require an implanted functional lifetime of several years, because otherwise surgeries would be needed too often. Transatlantic discussions within the ‘Decade of the Kidney^TM^’ initiative suggest 2–5 years between replacement surgeries could be a workable trade-off. Such an implantable HD filter should be much smaller in size while much higher in porosity than present fibre-based filters. Silicon wafer-based and/or other nanoscale manufacturing technologies might be key enablers to do this. In the past 60 years the chip industry did not only exponentially shrink the size and price of electronics, but also created chip-based microscopic scale electro-mechanic systems and microfluidics. These technologies may be applied in revolutionary HD filters.

Our literature search revealed two consortia using this approach: The USA-based ‘Kidney Project’ and the EU-based KIDNEW project (see Table 2, section ‘partially implantable’). Although both consortia apply very different methods to manufacture and coat their silicon nanopore membranes, their overall setup matches with the strongly simplified diagram in Fig. 4. Using an implanted HD filter with an extracorporeal dialysate circuit was accepted as fitting within the IEC definition of ‘haemodialysis’ during a meeting of IEC Technical Committee 62D/Maintenance Team 20 in June 2023 in Milan.

Whereas ‘the Kidney Project’ determines pore size with lithographic patterning, the KIDNEW projects utilizes molecular self-assembly to define pore size (which allows creating nanopores in denser patterns, increasing hydraulic porosity). KIDNEW additionally aims to make the implanted filter ‘smart’ by embedding chip-scale sensors and actuators (to enable embedded monitoring and cleaning of the filter while implanted) as well as wireless powering and bidirectional communication.

Both projects need an additional external device to provide the required dialysate. Preferably, this device should be miniaturized (e.g. by applying dialysate regeneration). Also, both projects target a modular stepwise approach: Besides the possibility for a partly implantable HD system, using an implanted SNM as HD filter, connectable to an additional extracorporeal dialysate circuit as shown in Fig. 4, they both also target (eventually) a fully implantable artificial kidney (IAK), consisting of an SNM HD filter and an additional bioreactor that mimics tubular functions. Although the IAK-concept goes beyond the definition of HD applied for the requested scope of this article, it would be a logical next step.

Note that, when comparing Figs. 3 and 4, the same dialysate regeneration technologies that enable a first miniaturization step can be re-used in combination with an implantable HD filter (but with even smaller weight and size, as the extracorporeal blood circuit is eliminated).

Figures 3 and 4 also illustrate the progressive ecological advantage of miniaturization.

## DISCUSSION

### QoL for patients on HD

Portable and wearable HD devices might not only improve QoL by increasing mobility, but also by improving the somatic domain of QoL. More frequent HD treatments (e.g. short daily HD or SDHD) or treatments with a longer duration and a lower flow rate (e.g. nocturnal HD) are associated with an improved health-related QoL [53, 54]. Portable and wearable devices allow patients to receive such treatments more easily, which could also improve their health-related QoL, as has been demonstrated for nocturnal HD and SDHD. A device without extracorporeal blood circuit will help promoting HHD (especially frequent nocturnal), as the risk of extracorporeal blood loss is eliminated.

It is important to mention that dialysis adequacy was seen to correlate appreciably with higher SF-36 scores in patients treated with PD [55]. Likewise, in conventional HD patients, Kt/V was strongly associated with increased QoL until 1.6, above which no further increase of quality of life occurred [56, 57]. Therefore, new HD modalities should not only focus on improving mobility, but also on improving treatment efficacy to maximize QoL.

All discussed trade-offs place natural constraints on design aspects such as weight, size, power, and dialysate consumption of the machine. Of course, adequacy is also a function of dialysis frequency and duration, which can generally be higher if mobility increases. This relaxes the constraints on machine and dialysate size and weight at the cost of spending more time on dialysis (which would in turn be more acceptable if time on dialysis would be less intrusive).

### Multi-year blood compatibility is crucial for implantable filters

No matter what type of material is used to create a nanoporous filter, to become implantable for several years, the blood-contacting surface must be made extremely biocompatible. Thus, the success of the approach as depicted in Fig. 4 will stand or fall with the realization of blood compatible coatings with superb specifications.

### Better funding mechanisms are needed

**Figure 5:**
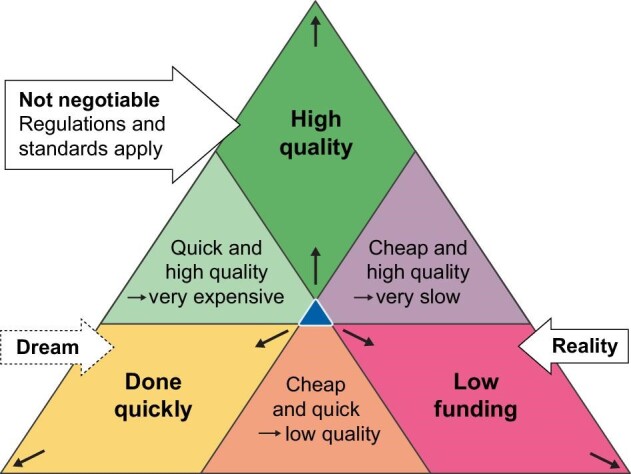
The ‘Triangle of Project Management’: You can pick only two of the three triangle points, the third one is automatically excluded. Note that developing complex medical devices anyhow requires considerable persistence. For a complex active medical device (such as a kidney replacement device), it typically takes ∼10 years to reach market approval, even if all parameters are optimized (depicted by the blue spot in the middle).

Translating a scientific discovery into a product that meets all minimum regulatory requirements on quality takes considerable time and money. The lower the funding, the longer development will take, illustrated by the triangle of project management in Fig. 5. Since governmental funding to stimulate R&D on better KRTs was practically stopped in the 1980s, the pace of innovation has been very slow and most HD patients are treated with machines like in Fig. 2. Yet, in the long run, governments could save considerable money with technologies such as those depicted in Figs 3 and 4. Firms (that typically need to provide quick return-on-investment to their shareholders) cannot easily cover such ‘long runs’. But they might be convinced to innovate by governmental co-funding arrangements (e.g. in combination with coopetition) [3].

## CONCLUSIONS

During the last decade, we have seen a revival of the strive to increase mobility of HD systems (the first wave started in the 1960s and nearly froze around 1980) [42, 43]. The COVID pandemic has highlighted the neglected advantages of home dialysis and demonstrated that extended remote care can work, which might help dialysis centres worldwide that are facing an increasing shortage of skilled workers.

Dialysate regeneration technologies can provide a first step towards increased mobility of HD systems. Technologies from the chip industry, which shrunk the size and price of electronics, microscopic scale electro-mechanic systems, and microfluidics, may enable further disruptive miniaturization of HD systems.

Interestingly, dialysate regeneration and chip technologies can be combined to (partly) implantable HD, offering elimination of the extracorporeal blood circuit (see Fig. 4), which is currently pursued by at least two projects (see Table 2).

Our kidneys make us mobile because they enable the disposal of strongly concentrated urea and other uremic toxins, without sacrificing loads of water. This article depicts a stepwise approach to realize HD systems that better mimic this kidney function.

Overall, truly portable, wearable, or even (partly) implantable HD devices probably will improve multiple domains of QoL once they become available on the market. Nevertheless, this can only be verified once such devices indeed become available to patients.

## Supplementary Material

sfae259_Supplemental_File

## Data Availability

No new data were generated or analysed in support of this research.
