# Supplementary material for: Transportable, portable, wearable and (partially) implantable haemodialysis systems: comparison of technologies and readiness levels
Source: Clin Kidney J. 2024 Aug 24;17(9):sfae259. doi: 10.1093/ckj/sfae259 (PMC11411285; doi:10.1093/ckj/sfae259)
Supplement: sfae259_Supplemental_File [file sfae259_supplemental_file.docx]

**Transportable, portable, wearable and (partially) implantable hemodialysis systems: comparison of technologies and readiness levels – SUPPLEMENTARY INFORMATION**

Fokko Wieringa^1,2,3,4^; Dian Bolhuis^2^; Henning Søndergaard^3,5^; Stephen Ash^6^; Cian Cummins^7^; Karin Gerritsen^2^; Jeroen Vollenbroek^1,2,8^; Tugrul Irmak^2,3^

1. IMEC, Holst Centre, Eindhoven, the Netherlands
2. UMCU, Utrecht, the Netherlands
3. EKHA WG3, Brussels, Belgium
4. IEC TC62D/MT20, Geneva, Switzerland
5. Danish Kidney Association, Copenhagen, Denmark
6. Purdue University, West Lafayette, Indiana, USA
7. IMEC, Leuven, Belgium
8. UTwente, Enschede, The Netherlands

**Details about applied Query**

With the aim to include scientific articles about transportable, portable, wearable or (partly) implantable hemodialysis systems (including engineering aspects and human factors), a structured literature search was conducted within the public domain PUBMED database, using the following query:

((portable[Title/Abstract]) OR (wearable[Title/Abstract]) OR (implantable[Title/Abstract])) AND ((hemodialysis[Title/Abstract]) OR (haemodialysis[Title/Abstract]) OR artificial kidney)) with a search time window from 01 Jan 2021 to 19 Dec 2023 (date of search).

This query revealed 159 results. For our inclusion/exclusion decision, we applied the definition of hemodialysis as given by the international standard IEC 60601-2-16, describing requirements for the safety and essential performance of hemodialysis devices, which defines hemodialysis as follows: *“Process whereby concentrations of water-soluble substances in a patient’s blood and an excess of fluid of a patient are corrected by bidirectional diffusive transport and ultrafiltration across a semi-permeable membrane separating the blood from the dialysis fluid”* [REF IEC 60601-2-16] [1]. Based upon the abstracts a first selection round was performed, which resulted in 22 direct inclusions (YES), 117 direct exclusions (NO), and 20 cases (MAYBE) where a final decision was made based on the whole article text. From these 20 “Maybe” results, 2 were additionally included, bringing the total number of included articles to 24.

The full content of all included articles was then searched for mentioning specific HD devices or projects targeted at realizing HD systems. Additional info about these devices/projects was collected via the internet, they were categorized regarding portability and TRL-values were assigned. The identified devices/projects were also grouped into 3 categories, using similar principles (Single-pass, Dialysate regenerating and partly implantable HD filter). Block diagrams to graphically summarize these 3 principles were created. The included articles were also analyzed regarding Medical, Technical, Economical, Usability and Quality-of-Life Trade-offs.

Excluded articles were clustered into distinct groups with various reasons for exclusion. Although the *content* of excluded articles was not further analyzed, the *themes* of the most dominant excluded groups formed a secondary input for the discussion section. Figure 2 shows a block diagram of the applied procedure.


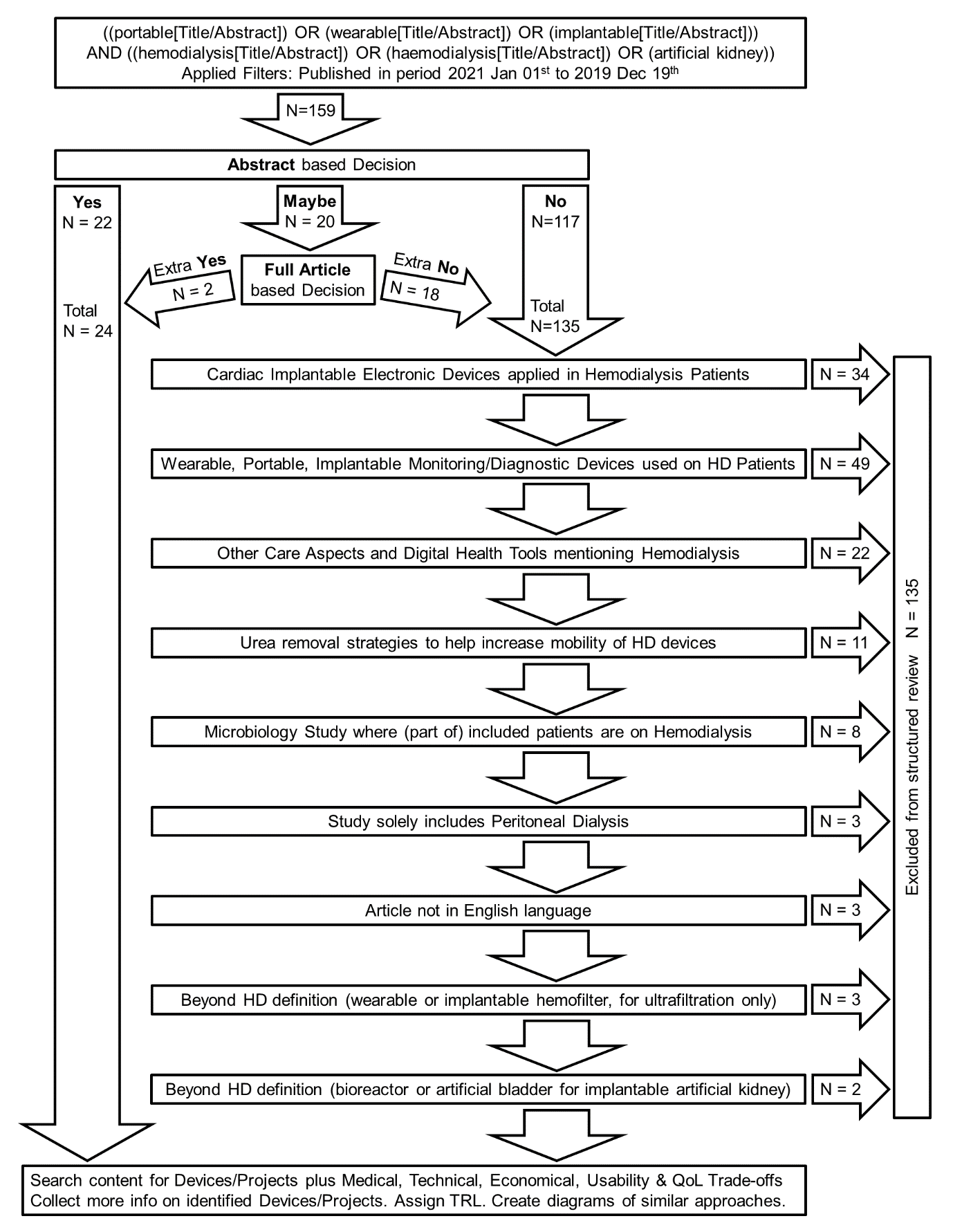
**Figure 2:** Query as applied onto the PUBMED database, plus the subsequently followed procedure for inclusion or exclusion of retrieved articles. Included articles were fully analyzed. Excluded articles were categorized into several distinct groups, and the themes of these groups provided some secondary input for the discussion section.

Where specific HD devices were named in the included articles, these devices were listed in Table 2 of the full article and an associated manufacturer website was added (if retrievable). Data about size, weight and functional principle were furthermore actively searched on the internet. Where no data was publicly available, the pertaining manufacturer was contacted to provide details, but further information was not always provided.

**References**

1. *International Standard IEC 60601-2-16. Particular Requirements for Basic Safety and Essential Performance of Haemodialysis, Haemodiafiltration and Haemofiltration Equipment*. 5th ed., 2018.
